# Crystal structure of 1-[(1-methyl-5-nitro-1*H*-imidazol-2-yl)meth­yl]pyridinium iodide

**DOI:** 10.1107/S2056989015001541

**Published:** 2015-01-28

**Authors:** Roumaissa Belguedj, Abdelmalek Bouraiou, Hocine Merazig, Ali Belfaitah, Sofiane Bouacida

**Affiliations:** a Unité de Recherche de Chimie de l’Environnement et Moléculaire Structurale, CHEMS, Université Constantine1, 25000 , Algeria; b Equipe de Synthèse de Molécules à Objectif Thérapeutique, Laboratoire des Produits Naturels d’Origine Végétale et de Synthèse Organique, Université Constantine 1, Constantine 25000, Algeria; c Département Sciences de la Matière, Faculté des sciences Exactes et Sciences de la Nature et de la Vie, Université Oum El Bouaghi, Algeria

**Keywords:** crystal structure, imidazole, pyridinium, iodide, hydrogen bonding

## Abstract

In the title salt, C_10_H_11_N_4_O_2_^+^·I^−^, the asymmetric unit consists of a pyridinium cation bearning a (1-methyl-5-nitro-1*H*-imidazol-2-yl)methyl group at the N position and an iodide anion. The imidazole ring is quasiplanar, with a maxiumum deviation of 0.0032 (16) Å, and forms a dihedral angle of 67.39 (6)° with the plane of the pyridinium ring. The crystal packing can be described as alternating zigzag layers of cations parallel to the (001) plane, which are sandwiched by the iodide ions. The structure features two types of hydrogen bonds (C—H⋯O and C—H⋯I), *viz*. cation–anion and cation–cation, which lead to the form ation of a three-dimensional network.

## Related literature   

For the synthesis and applications of imidazole derivatives, see: Upcroft & Upcroft (2001 ▸); Çelik & Ateş (2006 ▸); Boyer (1986 ▸); Olender *et al.* (2009 ▸); Gaonkar *et al.* (2009 ▸); Larina & Lopyrev (2009 ▸). For our previous work on this type of chemistry, see: Zama *et al.* (2013 ▸); Alliouche *et al.* (2014 ▸); Bahnous *et al.* (2012 ▸). For the synthesis of the title compound, see: Albright & Shepherd (1973 ▸).
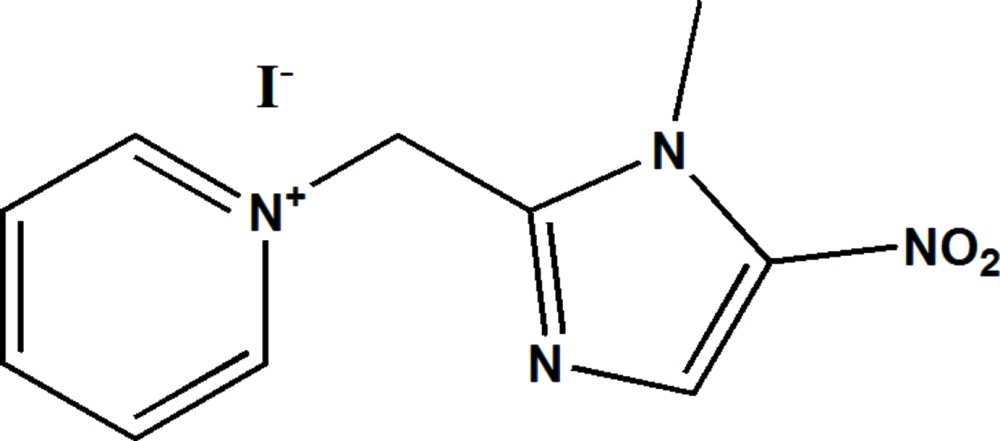


## Experimental   

### Crystal data  

C_10_H_11_N_4_O_2_^+^·I^−^
*M* *_r_* = 346.13
Monoclinic, 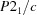

*a* = 11.035 (7) Å
*b* = 9.073 (6) Å
*c* = 12.859 (8) Å
β = 91.69 (2)°
*V* = 1286.8 (14) Å^3^
*Z* = 4
Mo *K*α radiation
μ = 2.49 mm^−1^
*T* = 295 K
0.14 × 0.12 × 0.11 mm

### Data collection  

Bruker APEXII diffractometer
Absorption correction: multi-scan (*SADABS*; Sheldrick, 2002 ▸) *T*_min_ = 0.615, *T*_max_ = 0.745
22502 measured reflections
6134 independent reflections
3669 reflections with *I* > 2σ(*I*)
*R*_int_ = 0.030

### Refinement  

*R*[*F* ^2^ > 2σ(*F* ^2^)] = 0.029
*wR*(*F* ^2^) = 0.067
*S* = 0.99
6134 reflections
155 parameters
H-atom parameters constrained
Δρ_max_ = 1.14 e Å^−3^
Δρ_min_ = −0.89 e Å^−3^

### 

Data collection: *APEX2* (Bruker, 2011 ▸); cell refinement: *SAINT* (Bruker, 2011 ▸); data reduction: *SAINT*; program(s) used to solve structure: *SIR2002* (Burla *et al.*, 2003 ▸); program(s) used to refine structure: *SHELXL97* (Sheldrick, 2015 ▸); molecular graphics: *ORTEP-3 for Windows* (Farrugia, 2012 ▸) and *DIAMOND* (Brandenburg, 2006 ▸); software used to prepare material for publication: *WinGX* (Farrugia, 2012 ▸).

## Supplementary Material

Crystal structure: contains datablock(s) I. DOI: 10.1107/S2056989015001541/hg5425sup1.cif

Structure factors: contains datablock(s) I. DOI: 10.1107/S2056989015001541/hg5425Isup2.hkl

Supporting information file. DOI: 10.1107/S2056989015001541/hg5425Isup3.cml

**Fig. 1.**
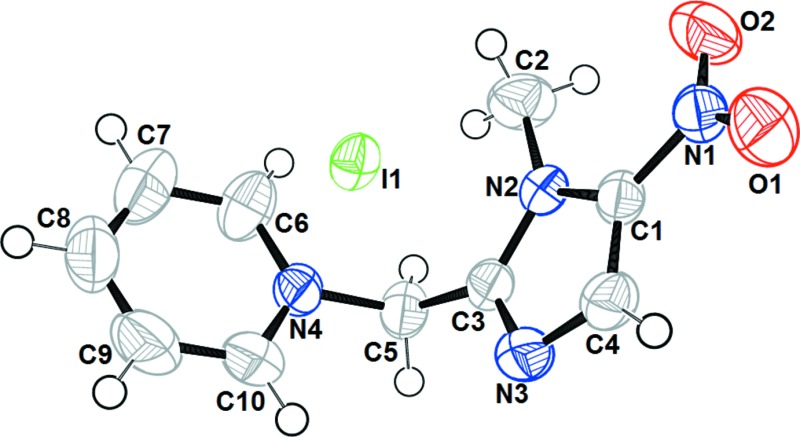
(Farrugia, 2012). The molecule structure of the title compound with the atomic labelling scheme. Displacement are drawn at the 50% probability level. H atoms are represented as small spheres of arbitrary radius.

**Fig. 2.**
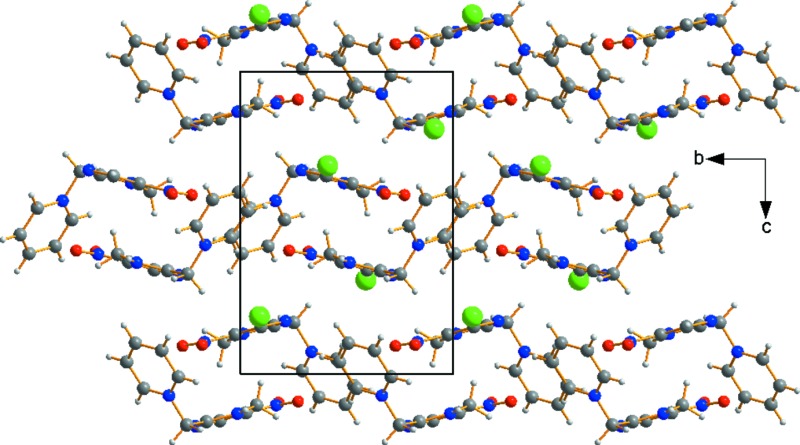
(Brandenburg, 2006). Alternating layers parallel to (001) plane of (I) sandwiched by iodide ions viewed via a axis

**Fig. 3.**
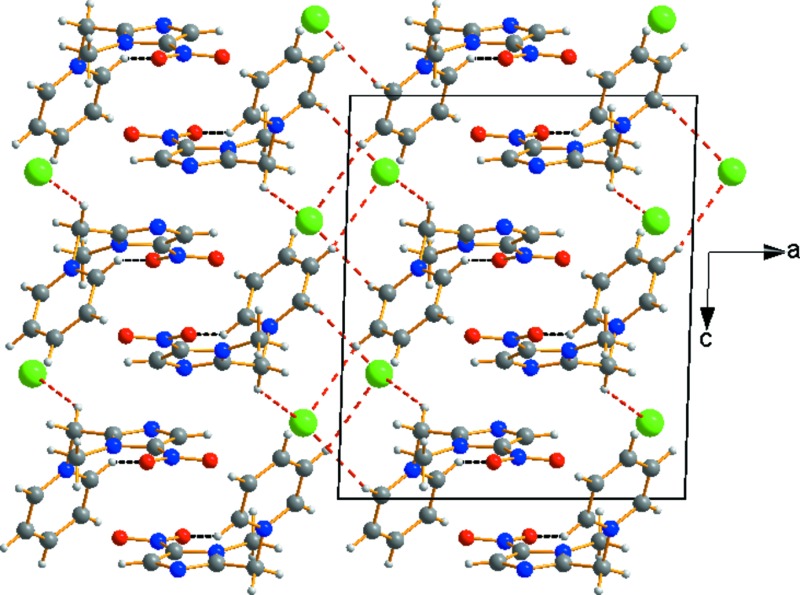
(Brandenburg, 2006). Crystal packing of (I) viewed via b axis showing hydrogen bond as dashed lines [C—H···I in red and C—H···O in black]

. DOI: 10.1107/S2056989015001541/hg5425fig1.tif
(Farrugia, 2012). The mol­ecule structure of the title compound with the atomic labelling scheme. Displacement are drawn at the 50% probability level. H atoms are represented as small spheres of arbitrary radius.

via a . DOI: 10.1107/S2056989015001541/hg5425fig2.tif
(Brandenburg, 2006). Alternating layers parallel to (001) plane of (I) sandwiched by iodide ions viewed *via a* axis

via b . DOI: 10.1107/S2056989015001541/hg5425fig3.tif
(Brandenburg, 2006). Crystal packing of (I) viewed *via b* axis showing hydrogen bond as dashed lines [C—H⋯I in red and C—H⋯O in black]

CCDC reference: 1045139

Additional supporting information:  crystallographic information; 3D view; checkCIF report

## Figures and Tables

**Table 1:** Hydrogen-bond geometry (, )

*D*H*A*	*D*H	H*A*	*D* *A*	*D*H*A*
C10H10O2^i^	0.93	2.51	3.138(3)	125
C5H5*A*I1^ii^	0.97	3.04	3.807(3)	137
C7H7I1^iii^	0.93	3.04	3.854(3)	147

Symmetry codes: (i) 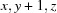; (ii) 
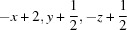
; (iii) 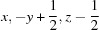.

## supplementary crystallographic information

### S1. Comment

1. Chemical Context

Nitroheterocyclic drugs have drawn a continuing interest over the years due to efficient use in the treatment of various anaerobic pathogenic bacterial and protozoal infections (Upcroft & Upcroft, 2001; Çelik & Ates, 2006). Nitroimidazole derivatives have been the subject of much research because of their properties. Depending on the nature and the position of substituents or the nitro group, the nitroimidazole derivatives can posses various pharmacological action (Boyer, 1986). Nitroimidazoles, such as metronidazole, misonidazole, ornidazole, secnidazole and etamidazole, are commonly used as therapeutic agents against a variety of protozoan and bacterial infections of humans and animals (Olender *et al.*, 2009; Gaonkar *et al.* 2009; Larina & Lopyrev 2009). In previous work, we have reported the synthesis and structure determination of some new heterocyclic compounds bearing a nitroimidazole entity (Zama *et al.*, 2013; Alliouche *et al.*, 2014; Bahnous *et al.*, 2012). Herein, we report the synthesis and single-crystal X-ray structure of 1-((1-methyl-5-nitro-1*H*-imidazol-2-yl)methyl)pyridinium iodide, (I).

2. Structural commentary

The molecule structure of (I), and the atomic numbering used, is illustrated in Fig. 1. The asymmetric unit of (I) consists of pyridinium cation bearing a 1-methyl-5-nitro-1*H*-imidazol-2-yl)methyl group at N position, and the iodide anion. The imidazol ring is quasiplanar with maxiumum deviation of 0.0032 (16) Å at C1 atom; and form dihedral angle of 67.39 (6)° with pyridinium ring. The crystal packing can be described by alternating layers in zigzag parallel to (001) plane of cations group, which are sandwiched by iodide ions (Fig. 2).

3. Supramolecular features

The crystal packing is mostly governed by classical hydrogen bonds (Fig. 3). Atoms C2, C5, C7, C10 and O2 of the cation participate in the formation of intramolecular [C—H···O and C—H···I] hydrogen bonds (Table 1). In this structure, we observe two types of hydrogen bonds, *viz*. cation-anion, cation-cation which form a three-dimensional network. The intramolecular hydrogen bond interactions C—H···O are also observed in cations moities. however the centroid to centroid distance between the phenyl rings are too long (4.430 (3) Å) for considering π-π interactions. These interactions link the molecules within the layers and also link the layers together and reinforcing the cohesion of the ionic structure.

### S2. Experimental

The 1-((1-methyl-5-nitro-1*H*-imidazol-2-yl)methyl)pyridinium iodide, I, was prepared from 1,2-dimethyl-5-nitro-1*H*-imidazole in presence of iodine and pyridine as solvent according to described procedure (Albright & Shepherd, 1973). The colorless crystals of the title compound used for the X-ray diffraction study were obtained from aqueous solution of I.

### S3. Refinement

The H atoms were localized on Fourier maps but introduced in calculated positions and treated as riding on their parent atom (C) with C—H = 0.93 Å (aromatic), C—H = 0.97 Å (methylene) and C—H = 0.96 Å (methyl) with *U*_iso_(H) = 1.2 or 1.5*U*_eq_(C).

### Crystal data

**Table:**

C_10_H_11_N_4_O_2_^+^·I^−^	*F*(000) = 672
*M**_r_* = 346.13	*D*_x_ = 1.787 Mg m^−^^3^
Monoclinic, *P*2_1_/*c*	Mo *K*α radiation, λ = 0.71073 Å
*a* = 11.035 (7) Å	Cell parameters from 6343 reflections
*b* = 9.073 (6) Å	θ = 2.8–29.3°
*c* = 12.859 (8) Å	µ = 2.49 mm^−^^1^
β = 91.69 (2)°	*T* = 295 K
*V* = 1286.8 (14) Å^3^	Prism, colorless
*Z* = 4	0.14 × 0.12 × 0.11 mm

### Data collection

**Table:**

Bruker APEXII diffractometer	6134 independent reflections
Radiation source: Enraf–Nonius FR590	3669 reflections with *I* > 2σ(*I*)
Graphite monochromator	*R*_int_ = 0.030
CCD rotation images, thick slices scans	θ_max_ = 36.5°, θ_min_ = 2.9°
Absorption correction: multi-scan (*SADABS*; Sheldrick, 2002)	*h* = −18→17
*T*_min_ = 0.615, *T*_max_ = 0.745	*k* = −14→14
22502 measured reflections	*l* = −21→21

### Refinement

**Table:**

Refinement on *F*^2^	Primary atom site location: structure-invariant direct methods
Least-squares matrix: full	Secondary atom site location: difference Fourier map
*R*[*F*^2^ > 2σ(*F*^2^)] = 0.029	Hydrogen site location: inferred from neighbouring sites
*wR*(*F*^2^) = 0.067	H-atom parameters constrained
*S* = 0.99	*w* = 1/[σ^2^(*F*_o_^2^) + (0.0275*P*)^2^] where *P* = (*F*_o_^2^ + 2*F*_c_^2^)/3
6134 reflections	(Δ/σ)_max_ = 0.006
155 parameters	Δρ_max_ = 1.14 e Å^−^^3^
0 restraints	Δρ_min_ = −0.89 e Å^−^^3^

### Special details

**Table:**

Geometry. All e.s.d.'s (except the e.s.d. in the dihedral angle between two l.s. planes) are estimated using the full covariance matrix. The cell e.s.d.'s are taken into account individually in the estimation of e.s.d.'s in distances, angles and torsion angles; correlations between e.s.d.'s in cell parameters are only used when they are defined by crystal symmetry. An approximate (isotropic) treatment of cell e.s.d.'s is used for estimating e.s.d.'s involving l.s. planes.
Refinement. Refinement of *F*^2^ against ALL reflections. The weighted *R*-factor *wR* and goodness of fit *S* are based on *F*^2^, conventional *R*-factors *R* are based on *F*, with *F* set to zero for negative *F*^2^. The threshold expression of *F*^2^ > σ(*F*^2^) is used only for calculating *R*-factors(gt) *etc*. and is not relevant to the choice of reflections for refinement. *R*-factors based on *F*^2^ are statistically about twice as large as those based on *F*, and *R*- factors based on ALL data will be even larger.

### Fractional atomic coordinates and isotropic or equivalent isotropic displacement parameters (Å^2^)

**Table:**

	*x*	*y*	*z*	*U*_iso_*/*U*_eq_	
C1	0.53170 (14)	−0.02548 (17)	0.12964 (12)	0.0288 (3)	
C2	0.75852 (17)	−0.0764 (2)	0.10429 (18)	0.0469 (5)	
H2A	0.8317	−0.0235	0.1217	0.07*	
H2B	0.755	−0.0965	0.031	0.07*	
H2C	0.7577	−0.1676	0.1421	0.07*	
C3	0.65440 (15)	0.15859 (18)	0.15888 (12)	0.0299 (3)	
C4	0.46650 (16)	0.09581 (18)	0.15569 (14)	0.0361 (4)	
H4	0.3826	0.0995	0.1603	0.043*	
C5	0.76681 (16)	0.25171 (19)	0.16974 (12)	0.0353 (4)	
H5A	0.7584	0.3202	0.227	0.042*	
H5B	0.8361	0.189	0.1855	0.042*	
C6	0.87603 (16)	0.2915 (2)	0.01029 (14)	0.0458 (4)	
H6	0.9214	0.2079	0.0268	0.055*	
C7	0.8991 (2)	0.3696 (3)	−0.07726 (17)	0.0563 (5)	
H7	0.9604	0.3395	−0.1206	0.068*	
C8	0.8319 (3)	0.4928 (3)	−0.10157 (16)	0.0633 (6)	
H8	0.8489	0.5479	−0.1603	0.076*	
C9	0.7398 (3)	0.5340 (2)	−0.03922 (18)	0.0698 (7)	
H9	0.6922	0.6156	−0.0562	0.084*	
C10	0.7181 (2)	0.4539 (2)	0.04885 (16)	0.0520 (5)	
H10	0.6556	0.481	0.092	0.062*	
N1	0.48460 (14)	−0.16821 (16)	0.10430 (11)	0.0361 (3)	
N2	0.65316 (11)	0.01303 (14)	0.13202 (9)	0.0278 (3)	
N3	0.54387 (13)	0.21140 (15)	0.17398 (11)	0.0377 (3)	
N4	0.78797 (12)	0.33517 (15)	0.07265 (10)	0.0323 (3)	
O1	0.37309 (12)	−0.17941 (15)	0.09568 (11)	0.0516 (3)	
O2	0.55464 (14)	−0.27106 (14)	0.09207 (13)	0.0594 (4)	
I1	1.107377 (10)	0.089936 (13)	0.189052 (9)	0.04248 (5)	

### Atomic displacement parameters (Å^2^)

**Table:**

	*U*^11^	*U*^22^	*U*^33^	*U*^12^	*U*^13^	*U*^23^
C1	0.0291 (8)	0.0313 (8)	0.0262 (7)	−0.0053 (6)	0.0025 (6)	0.0001 (6)
C2	0.0322 (10)	0.0468 (11)	0.0620 (12)	0.0067 (8)	0.0072 (9)	−0.0108 (9)
C3	0.0317 (8)	0.0315 (8)	0.0268 (7)	−0.0030 (6)	0.0042 (6)	0.0000 (6)
C4	0.0269 (8)	0.0405 (9)	0.0412 (9)	−0.0011 (7)	0.0070 (7)	−0.0027 (7)
C5	0.0376 (9)	0.0392 (9)	0.0291 (7)	−0.0098 (7)	0.0012 (7)	0.0012 (7)
C6	0.0322 (9)	0.0637 (12)	0.0416 (10)	−0.0018 (9)	0.0049 (8)	0.0067 (9)
C7	0.0492 (12)	0.0784 (15)	0.0417 (10)	−0.0130 (11)	0.0102 (9)	0.0030 (11)
C8	0.1028 (19)	0.0505 (12)	0.0368 (10)	−0.0304 (13)	0.0066 (11)	0.0034 (10)
C9	0.123 (2)	0.0316 (10)	0.0558 (13)	0.0119 (12)	0.0106 (14)	0.0052 (11)
C10	0.0778 (16)	0.0293 (8)	0.0498 (11)	0.0068 (10)	0.0161 (10)	−0.0007 (9)
N1	0.0418 (9)	0.0356 (7)	0.0311 (7)	−0.0086 (6)	0.0038 (6)	−0.0016 (6)
N2	0.0259 (7)	0.0309 (7)	0.0267 (6)	0.0000 (5)	0.0039 (5)	0.0005 (5)
N3	0.0354 (8)	0.0333 (7)	0.0449 (8)	−0.0005 (6)	0.0095 (6)	−0.0049 (6)
N4	0.0349 (8)	0.0312 (7)	0.0310 (6)	−0.0096 (6)	0.0028 (6)	−0.0037 (6)
O1	0.0413 (8)	0.0544 (8)	0.0589 (8)	−0.0164 (6)	−0.0041 (6)	−0.0063 (7)
O2	0.0606 (9)	0.0336 (7)	0.0847 (11)	−0.0003 (7)	0.0177 (8)	−0.0112 (7)
I1	0.03084 (7)	0.04881 (8)	0.04816 (8)	0.00945 (5)	0.00730 (5)	0.00972 (5)

### Geometric parameters (Å, º)

**Table:**

C1—C4	1.362 (2)	C5—H5B	0.97
C1—N2	1.385 (2)	C6—N4	1.338 (2)
C1—N1	1.429 (2)	C6—C7	1.360 (3)
C2—N2	1.470 (2)	C6—H6	0.93
C2—H2A	0.96	C7—C8	1.373 (3)
C2—H2B	0.96	C7—H7	0.93
C2—H2C	0.96	C8—C9	1.365 (4)
C3—N3	1.330 (2)	C8—H8	0.93
C3—N2	1.365 (2)	C9—C10	1.373 (3)
C3—C5	1.504 (2)	C9—H9	0.93
C4—N3	1.368 (2)	C10—N4	1.355 (2)
C4—H4	0.93	C10—H10	0.93
C5—N4	1.484 (2)	N1—O2	1.225 (2)
C5—H5A	0.97	N1—O1	1.236 (2)
			
C4—C1—N2	107.98 (14)	C7—C6—H6	120
C4—C1—N1	126.65 (15)	C6—C7—C8	119.9 (2)
N2—C1—N1	125.37 (14)	C6—C7—H7	120
N2—C2—H2A	109.5	C8—C7—H7	120
N2—C2—H2B	109.5	C9—C8—C7	119.7 (2)
H2A—C2—H2B	109.5	C9—C8—H8	120.1
N2—C2—H2C	109.5	C7—C8—H8	120.1
H2A—C2—H2C	109.5	C8—C9—C10	119.4 (2)
H2B—C2—H2C	109.5	C8—C9—H9	120.3
N3—C3—N2	112.53 (14)	C10—C9—H9	120.3
N3—C3—C5	122.78 (15)	N4—C10—C9	119.7 (2)
N2—C3—C5	124.69 (15)	N4—C10—H10	120.2
C1—C4—N3	109.28 (16)	C9—C10—H10	120.2
C1—C4—H4	125.4	O2—N1—O1	123.78 (15)
N3—C4—H4	125.4	O2—N1—C1	119.53 (15)
N4—C5—C3	110.99 (13)	O1—N1—C1	116.69 (15)
N4—C5—H5A	109.4	C3—N2—C1	104.61 (13)
C3—C5—H5A	109.4	C3—N2—C2	126.38 (14)
N4—C5—H5B	109.4	C1—N2—C2	128.85 (14)
C3—C5—H5B	109.4	C3—N3—C4	105.60 (14)
H5A—C5—H5B	108	C6—N4—C10	121.25 (16)
N4—C6—C7	119.9 (2)	C6—N4—C5	119.21 (15)
N4—C6—H6	120	C10—N4—C5	119.53 (15)
			
N2—C1—C4—N3	0.4 (2)	C5—C3—N2—C2	−3.1 (2)
N1—C1—C4—N3	−179.70 (15)	C4—C1—N2—C3	−0.60 (17)
N3—C3—C5—N4	−83.61 (19)	N1—C1—N2—C3	179.51 (14)
N2—C3—C5—N4	95.79 (18)	C4—C1—N2—C2	−176.19 (17)
N4—C6—C7—C8	−0.1 (3)	N1—C1—N2—C2	3.9 (3)
C6—C7—C8—C9	−1.8 (3)	N2—C3—N3—C4	−0.35 (19)
C7—C8—C9—C10	1.9 (4)	C5—C3—N3—C4	179.12 (15)
C8—C9—C10—N4	−0.1 (4)	C1—C4—N3—C3	−0.1 (2)
C4—C1—N1—O2	−173.00 (17)	C7—C6—N4—C10	2.1 (3)
N2—C1—N1—O2	6.9 (2)	C7—C6—N4—C5	−177.91 (17)
C4—C1—N1—O1	7.2 (2)	C9—C10—N4—C6	−2.0 (3)
N2—C1—N1—O1	−172.89 (15)	C9—C10—N4—C5	178.00 (19)
N3—C3—N2—C1	0.59 (17)	C3—C5—N4—C6	−105.28 (18)
C5—C3—N2—C1	−178.86 (14)	C3—C5—N4—C10	74.8 (2)
N3—C3—N2—C2	176.33 (16)		

### Hydrogen-bond geometry (Å, º)

**Table:**

*D*—H···*A*	*D*—H	H···*A*	*D*···*A*	*D*—H···*A*
C2—H2*C*···O2	0.96	2.50	2.861 (3)	102
C10—H10···O2^i^	0.93	2.51	3.138 (3)	125
C5—H5*A*···I1^ii^	0.97	3.04	3.807 (3)	137
C7—H7···I1^iii^	0.93	3.04	3.854 (3)	147

Symmetry codes: (i) *x*, *y*+1, *z*; (ii) −*x*+2, *y*+1/2, −*z*+1/2; (iii) *x*, −*y*+1/2, *z*−1/2.
