# Supplementary figures and images for: Crystal structure of 1-[(1-methyl-5-nitro-1H-imidazol-2-yl)meth­yl]pyridinium iodide
Source: Acta Crystallogr E Crystallogr Commun. 2015 Jan 28;71(Pt 2):o133–4. doi: 10.1107/S2056989015001541 (PMC4384567; doi:10.1107/S2056989015001541)

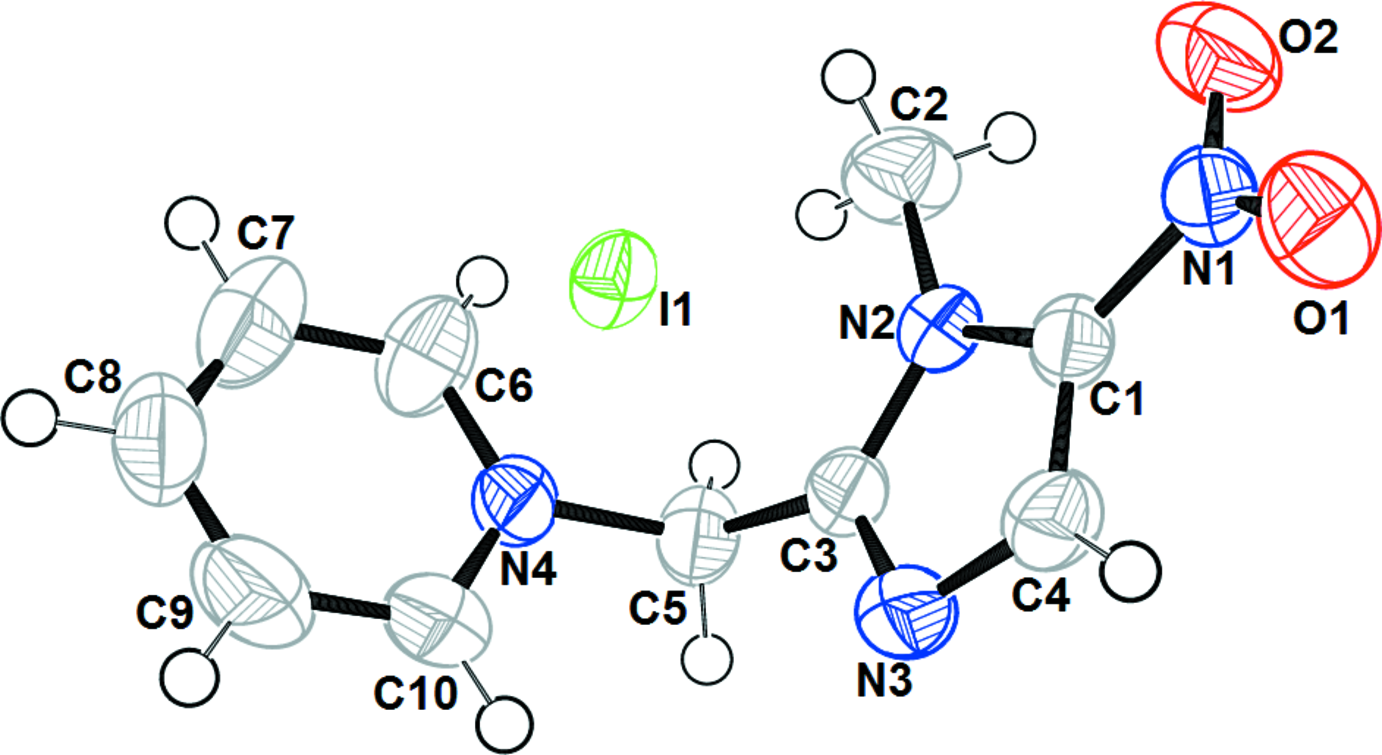

Supplement: Supplementary file 4 [file e-71-0o133-fig1.tif]

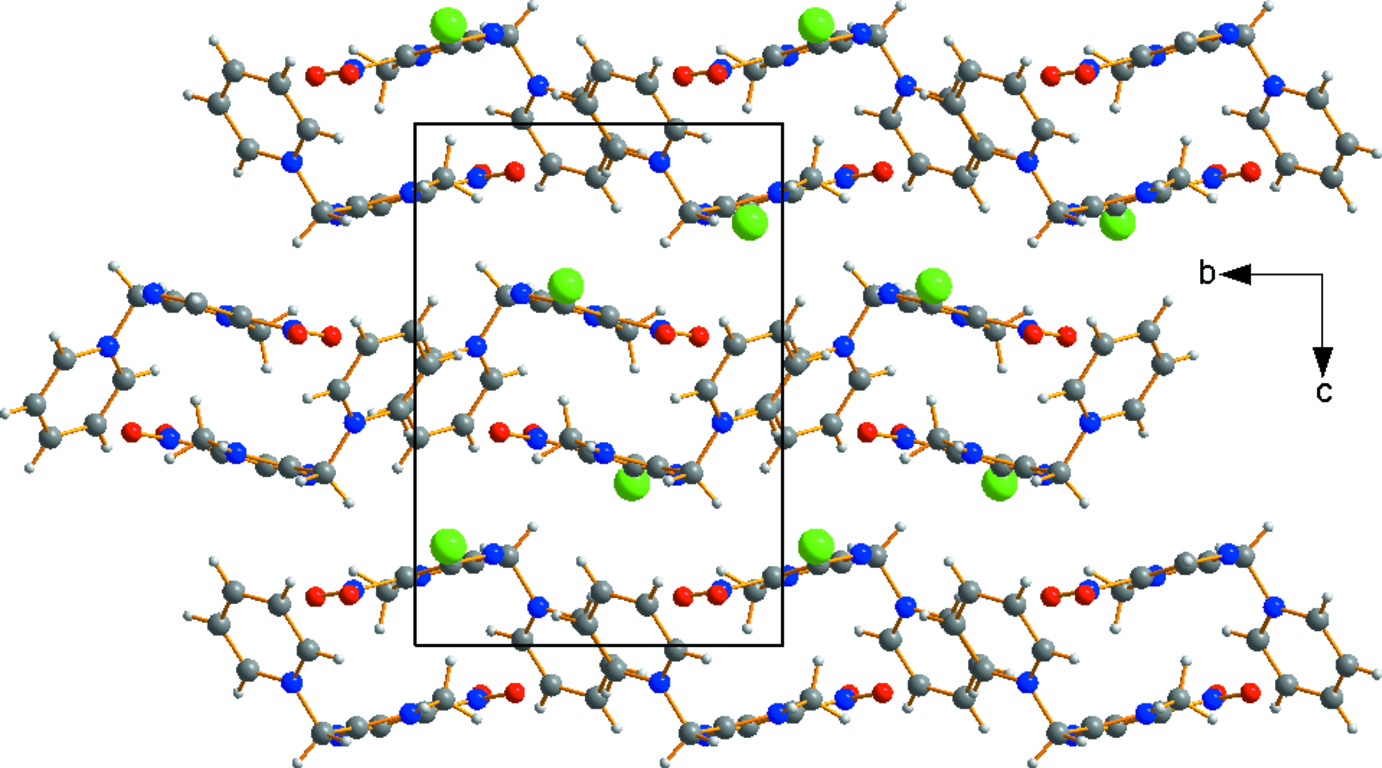

Supplement: Supplementary file 5 [file e-71-0o133-fig2.tif]

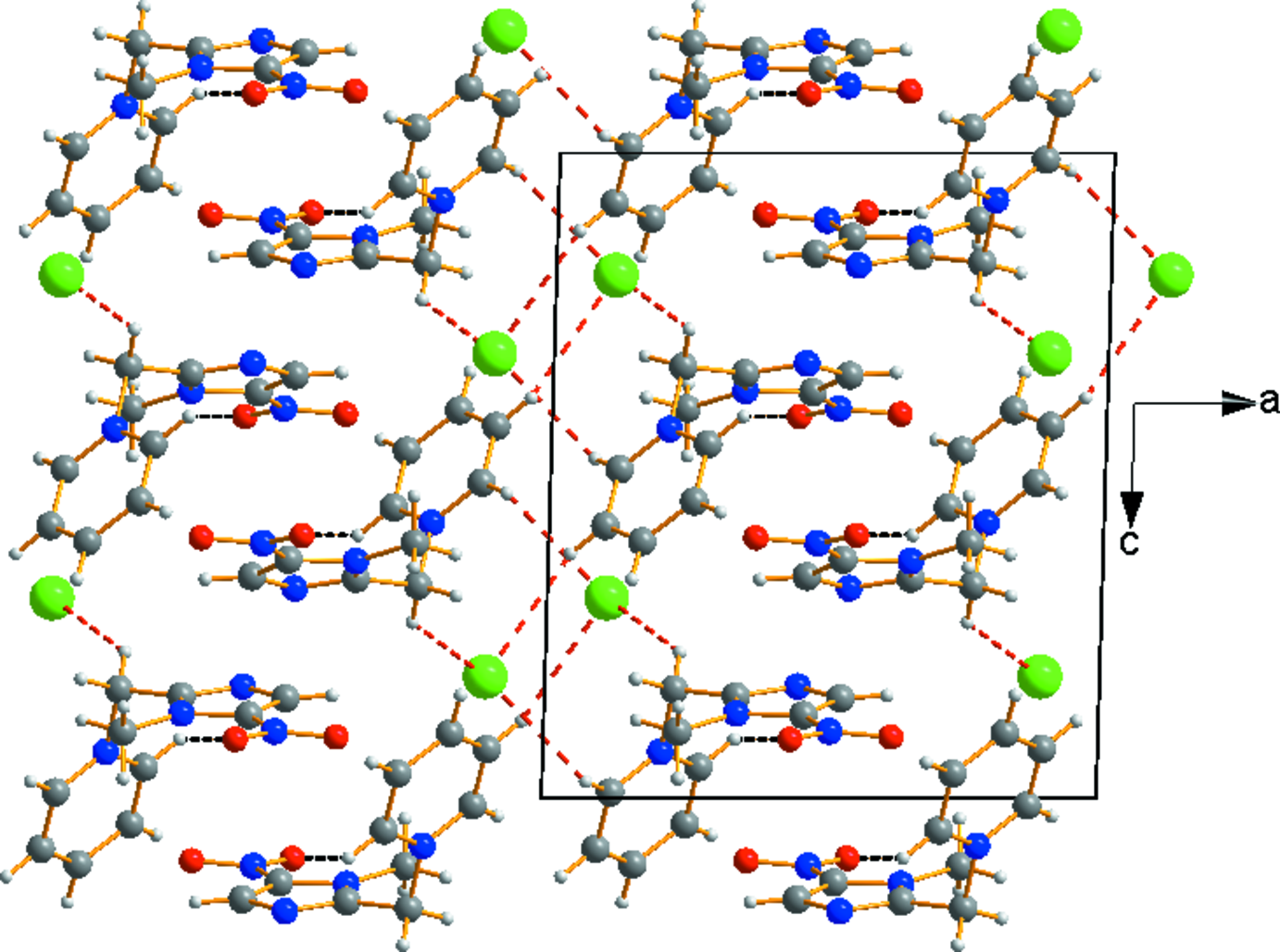

Supplement: Supplementary file 6 [file e-71-0o133-fig3.tif]
